# Dietary Supplementation for Attenuating Exercise-Induced Muscle Damage and Delayed-Onset Muscle Soreness in Humans

**DOI:** 10.3390/nu14010070

**Published:** 2021-12-24

**Authors:** Yoko Tanabe, Naoto Fujii, Katsuhiko Suzuki

**Affiliations:** 1 Faculty of Health and Sport Sciences, University of Tsukuba, Ibaraki 305-8574, Japan; fujii.naoto.gb@u.tsukuba.ac.jp; 2 Advanced Research Initiative for Human High Performance (ARIHHP), University of Tsukuba, Ibaraki 305-8574, Japan; 3 Faculty of Sport Sciences, Waseda University, Saitama 359-1192, Japan

**Keywords:** curcumin, tart cherry juice, beetroot juice, quercetin, isothiocyanate, oxidative stress, cytokines, inflammation, supplementation strategies, nutritional intervention, athletes

## Abstract

Dietary supplements are widely used as a nutritional strategy to improve and maintain performance and achieve faster recovery in sports and exercise. Exercise-induced muscle damage (EIMD) is caused by mechanical stress and subsequent inflammatory responses including reactive oxygen species and cytokine production. Therefore, dietary supplements with anti-inflammatory and antioxidant properties have the potential to prevent and reduce muscle damage and symptoms characterized by loss of muscle strength and delayed-onset muscle soreness (DOMS). However, only a few supplements are considered to be effective at present. This review focuses on the effects of dietary supplements derived from phytochemicals and listed in the International Olympic Committee consensus statement on muscle damage evaluated by blood myofiber damage markers, muscle soreness, performance, and inflammatory and oxidative stress markers. In this review, the effects of dietary supplements are also discussed in terms of study design (i.e., parallel and crossover studies), exercise model, and such subject characteristics as physical fitness level. Future perspectives and considerations for the use of dietary supplements to alleviate EIMD and DOMS are also discussed.

## 1. Introduction

Unaccustomed, strenuous high-intensity, or long-duration exercise can induce muscle damage, so-called exercise-induced muscle damage (EIMD). EIMD is characterized by a primary response as a result of mechanical stress that occurs during exercise and a secondary inflammatory response [1,2]. Mechanical force, especially that induced by eccentric contraction, leads to the primary response. More specifically, the overstretching and disruption of sarcomeres, followed by increased Ca^2+^ influx into the muscle cells, result in muscle passive tension and myofibrillar disruption [3]. These responses subsequently trigger secondary inflammatory responses, including the production of reactive oxygen species (ROS) and cytokines, by promoting the activation of transcription factors [e.g., nuclear factor-kappa B (NF-κB), mitogen-activated protein kinase (MAPK), and nuclear factor erythroid 2-related factor 2 (Nrf2)]. In addition, ROS and cytokines can be released from neutrophils and phagocytic macrophages [4,5]. ROS and exercise-induced inflammatory responses are essential for muscle repair, regeneration, and adaptation of redox signaling pathways; however, if left uncontrolled, they can result in cell infiltration into the damaged tissues, accelerating secondary muscle damage. Consequently, EIMD appears to cause several symptoms, such as loss of muscle function (e.g., force loss and reduced range of motion), delayed-onset muscle soreness (DOMS), and increased leakage of muscle proteins, such as creatine kinase (CK), myoglobin (Mb), and aspartate transaminase (AST), into the circulation. These symptoms can attenuate exercise performance. Therefore, it is important to minimize these symptoms to optimize athletic performance and conditioning as well.

Several nutritional strategies have been proposed to restore muscle function, relieve DOMS, and reduce inflammation after exercise. Based on the International Olympic Committee (IOC) consensus statement on dietary supplements and high-performance athletes, several dietary supplements, including creatine monohydrate, beta-hydroxybeta-methylbutyrate (HMB), omega 3-fatty acids, vitamin D, gelatin, vitamin C/collagen, and anti-inflammatory supplements, such as curcumin and tart cherry juice, may be effective in improving training capacity, recovery, muscle soreness, and injury management [6,7]. Among these supplements, anti-inflammatory supplements may attenuate DOMS [8,9]. Reduced DOMS may be important in sports activities, wherein soreness may impair performance in a subsequent bout of exercise [10]. Furthermore, it has been suggested that both inflammatory responses and ROS and free radicals produced during and following exercise may be involved in DOMS [11]. Thus, nutrition-based interventions targeting post-exercise inflammation and/or oxidative stress responses have received much attention. However, few supplements are considered effective [12].

When interpreting research outcomes, the study design needs to be carefully considered. The aforementioned IOC consensus statement notes that:

*“the gold standard for investigating the effects of supplements on sports performance is the prospective, randomized, controlled scientific trial, in which subjects are randomly allocated to receive either an experimental or placebo treatment (ideally in a double-blind manner) or crossed over to receive both treatments in counterbalanced order, under standardized conditions”*

[6].

The effects of supplements can be minimal, and it is therefore necessary to select a proper study design. In particular, since EIMD markers, including DOMS and CK exhibit large inter-individual differences [13,14], the study design needs to be carefully considered to detect small effects of supplements. In a parallel design, an appropriately large sample size should be selected. Furthermore, individual characteristics, especially fitness level and training history, should be carefully considered. On the other hand, the influence of inter-individual differences can be eliminated by employing a crossover design, though care should be taken to eliminate or minimize the repeated bout effect, which is the adaptation whereby a single bout of eccentric exercise protects against muscle damage from subsequent eccentric bouts. For example, after testing one limb for the first assessment, testing the contralateral limb for the second assessment would minimize the repeated bout effect. It should also be noted that when employing a contralateral exercise model, a longer washout period between the first and second measurements is required to minimize the contralateral repeated bout effect, which exerts a protective effect on the contralateral limb [15]. Moreover, testing trained individuals would be effective in minimizing repeated bout effect after exercise with submaximal intensity or sport-specific exercises [16,17].

In addition to study designs (i.e., parallel and crossover designs), the exercise model and physical fitness level should also be considered when interpreting supplement effects on EIMD. Regarding the exercise model, CK activity, which is an index of muscle fiber damage, was remarkably increased with a local muscle contraction model that accompanies eccentric exercises, such as drop jump, calf-raise, leg press, and arm curl. In addition, a greater degree of damage is associated with upper-limb exercise than with lower-limb exercise when matching the relative exercise intensity. However, elevations in inflammatory markers, such as cytokines (e.g., IL-1, IL-6, IL-10) and ROS, in response to local muscle contraction are relatively small, whereas whole-body exercises, such as endurance running and cycling, can greatly elevate these markers [18,19]. As for fitness level, trained individuals have less muscle damage than untrained individuals, despite both groups performing total-work matched exercise [20].

This review summarizes the supplementation strategies used to prevent and attenuate EIMD and DOMS in humans, with a focus on dietary supplements that are introduced in the IOC consensus statement and that have anti-inflammatory and/or antioxidant effects: (1) curcumin; (2) tart cherry juice; (3) beetroot juice, and (4) quercetin. Moreover, as an emerging new supplement, (5) isothiocyanate is also discussed. The potential effects of study design, exercise model, and physical fitness level are discussed. Additionally, future perspectives and considerations for the use of dietary supplements to alleviate EIMD and DOMS are also discussed.

## 2. Curcumin

Curcumin (*Curcuma longa* L.) is a natural polyphenolic substance extracted from turmeric. Curcumin has various physiological effects, and its underlying mechanisms have long been assessed in the field of clinical medicine. The major physiological effects of curcumin are anti-inflammatory and antioxidant effects [9], and responses occur through a decrease in the expression of pro-inflammatory genes. Therefore, curcumin has been shown to cure various diseases, including cancer, heart failure, and Alzheimer’s dementia [21,22,23]. In addition, curcumin exerts analgesic effects on acute and chronic pain by de-sensitizing the transient receptor potential vanilloid 1, an ion channel responsible for pain sensation, thereby reducing pain sensitivity [24,25]. Curcumin also regulates inflammatory cascades, such as NF-κB and Nrf2 pathways [26], and therefore possibly limits post-exercise inflammation, which subsequently reduces pain sensitivity and DOMS. As curcumin can act as a strong free radical scavenger [27], it may reduce secondary muscle damage. Therefore, in the IOC consensus statement, curcumin is classified as a nutritional supplement that may improve training capacity, recovery, muscle soreness, and injury management [6]. Regarding the antioxidant effect of curcumin after exercise, Takahashi et al. (2014) reported that curcumin ingestion lowers derivatives of reactive oxygen metabolites (d-ROMs), thioredoxin-1 (TRX-1), and glutathione (GSH), whereas it increases biological antioxidant potential (BAP) after treadmill walking or running at 65% VO_2max_ [28]. Chilelli et al. (2016) reported that the reduction of endogenous advanced glycation end products (AGEs) and malondialdehyde (MDA) was observed with curcumin ingestion in trained cyclists [29]. Notably, the bioavailability of curcumin was very low. Piperine, the active component of black pepper, can increase the bioavailability of curcumin when piperine and curcumin are co-ingested [30]. Microparticulation and surface treatment techniques have also been shown to enhance the bioavailability of curcumin [31,32]. Previous paralleled and crossover design studies that examined the effect of curcumin on EIMD and DOMS markers are summarized below (Table 1).

### 2.1. Paralleled Design Studies 

Drobnic et al. (2014) reported that moderately active individuals who ingested curcumin (4 days, 200 mg/day) exhibited lower IL-8 and DOMS in the lower limbs 2 h after a downhill running compared to the control condition, without differences in serum CK activity and oxidative stress markers [33]. Tanabe et al. (2019) reported that 7-day pre-exercise curcumin intake (180 mg/day) did not modulate maximum voluntary isometric contraction (MVIC) torque or range of motion (ROM) following eccentric contractions of the elbow flexors relative to the placebo intake. However, they reported that 3-day post-exercise curcumin intake (180 mg/day) improved the recovery of ROM and muscle soreness compared with the placebo intake [34]. This indicates that post-exercise curcumin intake may provide more beneficial effects in terms of reducing ROM and muscle soreness. More recently, Faria et al. (2020) demonstrated that long-term curcumin ingestion (29 days, 1500 mg/day) resulted in a lower Mb concentration and a greater increase in IL-10 following the half-marathon race compared with the placebo ingestion [35], suggesting that some anti-inflammatory mechanisms were induced by curcumin in EIMD.

### 2.2. Crossover Design Studies 

Nicol et al. (2015) reported that curcumin ingestion (5 days, 5 g/day) decreased CK and IL-6 concentrations after leg press resistance exercise relative to the placebo ingested condition [36]. In this study, curcumin ingestion resulted in reductions in pain and an improvement in muscle performance, as assessed by an increase in jump height during single-leg squats 24 and 48 h after eccentric single-leg press exercise in physically active individuals. Tanabe et al. (2015) demonstrated that curcumin intake 1 h before and 12 h after eccentric exercise of the elbow flexors (each 150 mg) attenuated the reduction in MVIC torque and an increase in serum CK activity in untrained men, without modulating IL-6, tumor necrosis factor-α (TNF-α), and other markers (DOMS, ROM, and upper-arm circumference) [31]. Delecroix et al. (2017) assessed responses in elite rugby players, demonstrating that 4-day (2 days before and 2 days after exercise) curcumin ingestion (6 g curcumin and 60 mg piperin) attenuated reductions in power output during repetitive sprint in comparison to the placebo condition, with no effect on DOMS and CK [30]. Tanabe et al. (2019) reported that pre-exercise curcumin ingestion (7 days, 180 mg/day) attenuated increases in IL-8 after elbow flexor exercise, but this response was not observed when curcumin was administered post-exercise (7 days, 180 mg/day) compared to the placebo ingestion condition. However, post-exercise curcumin ingestion attenuated elevations in CK, muscle soreness, and reductions in MVIC torque of the elbow flexors and ROM of the elbow joint [37]. Markers of oxidative stress (i.e., d-ROMs) and oxidative elimination ability (i.e., BAP) did not change over the course of the experiment.

### 2.3. Summary

Irrespective of paralleled [33,34,35] or crossover [30,31,36,37] design, previous studies demonstrated that curcumin ingestion attenuates some inflammatory responses, as assessed by IL-6, IL-8, TNF-α, and/or IL-10, regardless of exercise modalities (e.g., aerobic or resistance exercise) [33,35,36,37]. Based on previous studies (Table 1), starting curcumin intake at least 2 days prior to exercise appears to be necessary for reducing inflammatory responses, regardless of the type of exercise. Regarding the antioxidant effect on responses associated with muscle-damaging exercise, only two studies are available (one for parallel study design [33] and the other for crossover design [37]), suggesting that no measurable antioxidant effect of curcumin is detected after downhill running [33] and upper arm eccentric exercise [37]. Regarding DOMS, due to the analgesic effects of curcumin [38], positive effects were observed in the parallel design [33,34], whereas the results were equivocal for the crossover design, such that two studies reported positive effects [36,37] while the other two reported no effects [30,31]. Given that a previous study reported no effects in elite rugby players [30], the effect of curcumin on attenuating DOMS might be diminished in elite or highly trained athletes. Moreover, DOMS was alleviated only when curcumin was administered consecutively after exercise [34,37]. Thus, continuous ingestion of curcumin during the post-exercise period might be necessary to attenuate DOMS. Regarding the performance markers, positive effects were observed in parallel design [34] and all crossover design [30,31,36,37] studies, including MVIC [31,37], jump height [36], and sprint [30]. However, the effect of curcumin on ROM is ambiguous [31,34,37], and no effect has been reported on swelling [31,36,37].

## 3. Tart Cherry or Tart Cherry Juice

Tart cherry juice, made from tart Montmorency cherries, contains numerous phytochemicals, including anthocyanins and flavonoids [39]. Anthocyanins with high antioxidant content are thought to scavenge ROS and limit ROS production [40]. Tart cherry juice has been shown to lower the risk of diabetes and cardiovascular diseases [41]. Anthocyanins and flavonoids in the tart cherry juice can inhibit enzyme activities, such as cycrooxigenage-2 (COX-2) and phospholipase A2, and may ultimately exhibit anti-inflammatory effects [42,43]. Therefore, tart cherry juice may better maintain the inflammatory response and redox balance, thereby improving recovery following strenuous exercise [44]. The IOC consensus statement mentions that the anti-inflammatory effects of tart cherry juice may be beneficial in promoting recovery, although benefits may be sport/training-specific. The dose of tart cherry juice needed to promote recovery appears to be 250–350 mL (30 mL if concentrated) twice daily for 4–5 days before an athletic event or for 2–3 days afterwards. Moreover, the amount of tart cherry juice intake, especially total phenolic content, is a key factor that determines its effects. A recent review article concluded that enhancing recovery following muscle damage via antioxidant and anti-inflammatory mechanisms may require >1000 mg polyphenols per day for 3 or more days prior to and following exercise [45]. Review articles concluded that tart cherry juice may attenuate inflammatory and oxidative responses to EIMD, ultimately accelerating faster recovery after bouts of muscle-damaging exercise [46,47]. Previous paralleled and crossover design studies that examined the effect of tart cherry juice on EIMD and DOMS markers are summarized below (Table 2).

### 3.1. Paralleled Design Studies 

Howatson et al. (2010) reported that 236 mL of tart cherry juice ingested twice per day for 8 days attenuated decreased MVIC and inflammatory markers [IL-6, C-reactive protein (CRP)] after marathon running in recreational runners [39]. Moreover, total antioxidant status (TAS) was greater and oxidative stress, as assessed by thiobarbituric acid reactive species (TBARS), was lower in the tart cherry juice group than in the placebo group. Bell et al. (2016) reported that individuals who ingested tart cherry juice (8 days, 30 mL twice/day) exhibited lower IL-6 and DOMS in the lower limbs and a faster recovery of knee extensor MVIC, CMJ, and agility after the Loughborough intermittent shuttle test (LIST) compared to the control group, without differences in serum CK activity and oxidative stress marker (LOOH) in semi-professional male soccer players [48]. Recently, Quinlan et al. (2019) reported that tart cherry juice (8 days, 30 mL twice/day) accelerated the recovery of CMJ, 20-m sprint, and MVIC of knee extensors following LIST compared to the placebo conditions in team sports players (football, hockey, or netball sports) [49]. In contrast, Lamb et al. (2019) demonstrated that 9-day tart cherry juice ingestion (30 mL twice/day) had no effects on MVIC, DOMS, CK, or ROM after the elbow flexors of the non-dominant arm exercise relative to the placebo drink ingestion group in non-resistant trained men [50].

### 3.2. Crossover Design Studies

Connolly et al. (2006) reported that 355 mL of tart cherry juice twice/day (for 8 days) ingestion attenuated decreased MVIC and DOMS after eccentric exercise of the elbow flexors in college students [51]. On the other hand, in well-trained male, Bowtell et al. (2011) reported that 30 mL twice/day (for 10 days) ingestion attenuated reductions in MVIC and increases in protein carbonyls without affecting CK, CRP, DOMS, and other antioxidant status markers (nitrotyrosine and TAS) after knee extensions [52]. Meanwhile, in professional athletes, Morehen et al. (2020) reported that 8-day tart cherry juice consumption (30 mL twice/day) had no effect on cytokine responses (IL-6, IL-8, and IL-10), DOMS, or jump performance (CMJ and drop jump) after professional league matches in rugby players compared with the placebo [53]. Similarly, Abbott et al. (2020) reported no effects of tart cherry juice ingestion (2 shots × 30 mL, before and after the match, 12 and 36 h after the match) on muscle function (CMJ and reactive strength index), self-reported well-being, and muscle soreness after a 90-min soccer match in male professional soccer players in comparison to the control group [54]. 

### 3.3. Summary

Irrespective of the study design, mixed results have been reported regarding the effects on exercise performance. Among these, positive effects were reported for the types of markers assessed in MVIC [39,48,49,51,52], CMJ [48,49], and sprint [49]. As for DOMS, most studies reported no effects in both parallel [39,49,50] and crossover [52,53,54] studies; however, some studies also reported positive effects [48,51], regardless of the study design. The effects of tart cherry on inflammatory (IL-6, IL-8, IL-10, and CRP) and oxidative stress (TAS, TBARS, LOOH, nitrotyrosine, and protein carbonyls) markers are not universal in parallel studies [39,48,49]. However, in crossover studies, no effect was detected on inflammatory markers [52,53], but protein carbonyls, an oxidative stress marker, were reduced [52]. No effect of tart cherry juice was consistently observed on muscle damage markers in blood (CK and LDH), regardless of a parallel [39,48,49,50] or crossover [52] study design. Notably, two crossover studies reported no effect on professional soccer [54] or rugby [53] players. Thus, the effect of tart cherry on indices associated with EIMD and DOMS may be diminished in elite or highly trained individuals. In addition, the aforementioned crossover studies assessed responses before and after match play, which would mediate less muscle damage relative to laboratory-based exercise loads, such as eccentric exercise. Therefore, the effect of tart cherry juice might be hardly detectable under conditions where sport-specific exercises, such as soccer and rugby match, are employed. In the future, studies will be needed to delineate the optimal amount and duration of tart cherry juice intake in terms of the effect on DOMS in athletes.

## 4. Beetroot Juice

Red beetroot (*Beta vulgaris rubra*) is a functional food that contains high levels of nitrate and other phytochemicals, including bioactive compounds, such as betalain, ascorbic acid, carotenoids, phenolic acids, and flavonoids. Chronic and acute beetroot juice supplementation has been shown to improve blood pressure control, vascular function, and renal health [55]. Nitrate in the beetroot juice increases nitric oxide (NO) bioavailability, which subsequently improves vascular function, mitochondrial efficiency, glucose homoeostasis, and muscle contractility of type II fibers, thereby improving exercise performance [56], especially endurance exercise performance (in the range of 5–30 min) [57]. Therefore, nitrate is classified as a nutritional supplement that directly improves sports performance in the IOC consensus statement [6]. Moreover, beetroot juice may improve sprint and cognitive performance [58]. As for the timing of ingestion, beetroot juice needs to be ingested at least 90 min prior to the event [59]. On the other hand, betalain, the most potent antioxidant molecule found in beetroot, is thought to attenuate ROS scavenging and upregulate endogenous antioxidant enzymes. Nitrites have also been shown to inhibit radical formation and ROS production. Moreover, betalain is responsible for its analgesic effects via an anti-inflammatory related mechanism [60]. Therefore, beetroot juice is expected to accelerate the recovery of muscle damage by directly or indirectly reducing exercise-induced ROS production and DOMS. Previous paralleled and crossover design studies that examined the effect of beetroot juice on EIMD and DOMS markers are summarized below (Table 3).

### 4.1. Paralleled Design Studies 

Clifford et al. assessed the effect of beetroot juice on EIMD and DOMS in a series of studies [61,62,63,64]. They examined the acute effect of beetroot juice [a higher dose (250 mL, ~250 mg of nitrate) and a lower dose (125 mL, the same composition as the higher dose beetroot juice, but provided half the dose)], and an isocaloric placebo drink (250 mL, with negligible nitrate content) consumed immediately (×3 bottles), 24 h (×2 bottles), and 48 h (×2 bottles) after 100-drop jumps in 30 physically active men. In this study, regardless of dosage, beetroot juice supplementation attenuated DOMS at 24, 48, and 72 h post-exercise, and reduced CMJ performance 72 h post-exercise. However, there were no significant differences in any of the cytokines assessed (IL-6, TNF-α, and IL-8) regardless of the amount of beetroot juice ingested [62]. Using the same drop jump protocol, a subsequent study by Clifford and colleagues reported that DOMS evaluated by pressure pain-threshold following 100-drop jumps was attenuated by beetroot juice ingestion (~210 mg of nitrate) compared to NO_3_^−^ dose-matched sodium nitrate drink or placebo drink. Therefore, beetroot juice supplementation is more effective than sodium nitrate in attenuating DOMS associated with EIMD. The authors concluded that phytonutrients other than nitrate, such as betalains and phenolics, or interactions between them (or with nitrate), are likely responsible for its analgesic effects [64]. Clifford et al. also examined the effect of beetroot juice using two bouts of repeated-sprint exercise models [63]. In this study, beetroot juice ingestion (2 × 250 mL/day, ~251 mg/bottle of nitrate, 4 days) attenuated DOMS evaluated by pressure pain threshold and reductions in CMJ performance relative to placebo ingestion condition. However, beetroot juice did not affect the indirect oxidative stress markers (LOOH and protein carbonyls) and a direct marker of free radical production (ascorbyl free radical). Moreover, the same research group reported no effect of beetroot juice on responses after the marathon race [61]. Specifically, total blood leukocyte, neutrophil, and monocyte counts peaked immediately after marathon race, and responses did not return to pre-marathon values at day 2 post-marathon in both beetroot juice [total 6 bottles (250 mL/bottle, ~210 mg of nitrate) 3 days post-marathon] and an isocaloric placebo groups. Furthermore, the responses of cytokines (IL-6, IL-8, and TNF-α), CK, AST, CRP, and muscle soreness were not different between the two groups. Thus, beetroot juice does not appear to modulate inflammation or reduce muscle damage after prolonged endurance exercise.

### 4.2. Crossover Design Studies

Van Hoorebeke et al. (2016) reported that betalain-rich supplementation (100 mg/day for 6 days before the exercise trials and 50 mg on day 7), containing no sugars or nitrates, improved 5-km time trial performance and attenuated elevations in LDH from baseline compared to placebo ingestion in young competitive runners [65]. Montenegro et al. (2017), under similar supplementation conditions (dose and ingestion timing), reported that betalain-rich supplementation improved 10-km running time trial performance and 5-km time trial performance (performed 24 h after the 10-km time trial), as well as attenuating increased CK in competitive male and female triathletes [66]. Daab et al. (2020) reported that 7-day beetroot juice supplementation (2 × 150 mL/day, 3 days pre-exercise, day of test, and 3 days after intermittent damaging exercise) reduced muscle soreness and LDH, and improved the recovery of muscle function (CMJ, MVIC) after intermittent damaging exercise in soccer players [67].

Recently, although the order of intervention was not counterbalanced, long-term (4 weeks) beetroot juice supplementation (26 g/day freeze-dried beetroot) increased lipid peroxidation (i.e., MDA) in elite fencers. This result is unexpected because beetroot juice can decrease oxidative stress. The authors of a previous study speculated that beetroot juice consumption might have increased physical activity, ultimately increasing oxidative stress. Additionally, a significant increase in VO_2max_ was observed after ingestion of beetroot juice without attenuated muscle damage markers, such as CK and LDH [68]. 

**Table 3:** Effect of beetroot juice on EIMD and DOMS markers.

Reference (Year)	Population	Supplementation		Exercise	Outcome				
		Dose	Duration		Blood Damage Maker	Functional Performance Marker	DOMS, Pain	Inflammatory Marker	Oxidative Stress Marker
*Paralleled design studies*									
Clifford et al. (2016) [62]	Recreationally active males	H-BT: 250 mL of BTJ	3 d Ex-d (×3 servings), 24 h (×2 servings) and 48 h (×2 servings) post-Ex	Drop jumps	CK: ✕	MVIC: ✕	PPT: ◯ (H-and L-BT)	IL-6, TNF-α, IL-8: ✕	
		L-BT: 125 mL of BTJ							
						CMJ: ◯ (H-BT)			
		PLA: 250 mL of placebo							
Clifford et al. (2017) [64]	Recreationally active males	BTJ: 250 mL of BTJ	3 d Ex-d (×3 servings), 24 h (×2 servings) and 48 h (×2 servings) post-Ex	Drop jumps	CK: ✕	MVIC, CMJ: ✕	PPT: ◯ (BLJ)	CRP: ✕	
		SN: 250 mL of sodium nitrate							
		PLA: 250 mL of placebo							
Clifford et al. (2016) [63]	Male team-sports players	500 mL of BTJ or a placebo	4 d (Ex-d, 24, and 48 h post-RST1 and 30-min post-RST2)	RST1: (first Ex)	CK: ✕	MVIC, sprint: ✕	PPT: ◯	CRP: ✕	LOOH, PC, A•−: ✕
				RST2: (second Ex)		CMJ, reactive strength index: ◯			
Clifford et al. (2017) [61]	Runners, males and females	250 mL of BTJ or a placebo	3 d Ex-d (×3 servings), 24 h (×2 servings) and 48 h (×1 serving) post-Ex	Marathon	CK, AST: ✕	MVIC, CMJ: ✕	VAS: ✕	IL-6, TNF-α, IL-8, CRP: ✕	
*Crossover design studies*									
Van Hoorebeke et al. (2016) [65]	Competitive male runners	Betalain-rich concentrate capsule or placebo	7 d (D 1–6: 50 mg, twice/d; D 7: 50 mg pre-Ex	30 min of treadmill running followed by a 5-km TT	LDH (from baseline): ◯	HR, RPE, lactate concentration,5-km TT duration: ◯	VAS: ✕		
					CK, LDH: ✕	Fatigue: ✕			
Montenegro et al. (2017) [66]	Triathletes, males and females	Betalain-rich concentrate capsule or placebo	7 d (D 1–6: 50 mg, twice/d; D 7: 50 mg pre- Ex	40 min of cycling followed by a 10-km running TT	CK: ◯	10-km TT duration, 5-km TT duration, Fatigue: ◯	VAS: ✕		
					LDH: ✕	HR average, RPE: ✕			
Daab et al. (2020) [67]	Male soccer players	150 mL BTJ or placebo, twice/day	7 d (3 d pre-Ex, Ex-d and 3 d post-Ex)	LIST	CK: ◯	CMJ, MVIC, sprint: ◯	VAS: ◯	CRP: ✕	
					LDH: ✕	Squat jump: ✕			
Kozłowska et al. (2020) [68]	Elite fencers, males and females	Dietary recommendations with 26 g/day of freeze-dried BTJ or without BTJ	4 weeks	Fencing and general training	CK, LDH: ✕	VO_2max_: ◯		IL-6: ✕	MDA, GPx-1: ◯
									GPx-3, AOPP, 8-oxodG: ✕

◯, effective; ✕, ineffective; DOMS, delayed-onset muscle soreness; IL-6, interleukin-6; IL-8, interleukin-8; TNF-α, tumor necrosis factor-α; CRP, C-reactive protein; CK, creatine kinase; AST, aspartate aminotransferase; LDH, lactate dehydrogenase; MVIC, maximal voluntary isometric contraction; CMJ, counter movement jump; VAS, visual analogue scale; PPT, pressure pain threshold; RPE, rate of perceived exertion; HR, heart rate; PC, protein carbonyls; GPx-1, glutathione peroxidase-1; GPx-3, glutathione peroxidase-3; LOOH, lipid hydroperoxides; MDA, malondialdehyde; AOPP, advanced oxidation protein product; 8-oxodG, 8-oxo-7.8-dihydro-2′-deoxyguanosine; A•−, plasma ascorbate free radical; PLA, placebo; BTJ, beetroot juice; SN, sodium nitrate; Ex, exercise; RST, repeated sprint test; VO_2max_, volume oxygen consumption maximum; TT, time trial; LIST, Loughborough intermittent shuttle test.

### 4.3. Summary

One parallel study showed no effect on oxidative stress [63], whereas one crossover study showed that beetroot juice increased oxidative stress [68]. Previous studies demonstrated that beetroot juice had no effect on inflammatory responses (IL-6, IL-8, CRP, and TNF-α) following exercise modalities (sprint, plyometric, intermittent, and endurance exercise) regardless of parallel [61,62,63,64] or crossover [67,68] study design. From these results, it is thought that the antioxidant and anti-inflammatory effects of beetroot juice after exercise may be minimal based on the blood indices. However, blood markers may not directly reflect muscle conditions. Therefore, it remains to be elucidated whether beetroot juice modulate oxidative and inflammatory responses in muscles. Muscle pain, as measured by changes in pressure pain threshold, is alleviated by beetroot juice supplementation in a parallel study design; however, these studies by Clifford et al. did not employ a crossover study design. Muscle damage markers in blood, such as CK and AST, were not affected by beetroot juice intake in all parallel design studies wherein a drop jump, sprint, and marathon race were employed [61,62,63,64]. However, the CK and LDH levels were elevated following intense running or cycling, which was attenuated by beetroot juice intake in some crossover design studies [65,66,67]. Together, these findings suggest that the effect of beetroot juice on blood muscle damage indices is highly dependent on the study design, and crossover design studies suggest that beetroot juice may reduce muscle damage caused by endurance exercise. Meanwhile, most crossover studies have found that beetroot juice supplementation promotes faster recovery of sprint [67], CMJ [67], MVIC [67], time trial duration [65,66] and VO_2max_ [68] after muscle damage exercise. Furthermore, approximately half of the previous studies employing parallel designs detected positive effects on the aforementioned performance indices [62,63]. A possible reason for this improved performance may be related to increased electromyography amplitude during maximal isometric voluntary contractions, the improvement of neuromuscular efficiency, and improved cardiorespiratory performance, as suggested in a previous study [69,70], independent of muscle damage conditions. Therefore, the consumption of beetroot juice may be effective in improving the performance even under EIMD conditions.

## 5. Quercetin

Quercetin is a plant flavonoid found in green tea, red and white onions, apples, peppers, blueberries, and dark green vegetables. Quercetin has antioxidant and anti-inflammatory properties, as well as cardioprotective, anticancer, and hepatoprotective effects [71]. In addition, quercetin has antipathogenic activities [72] which may influence immune system and resistance to pathogens. Therefore, quercetin is classified as a nutritional supplement for immune health in athletes in the IOC consensus statement [6]. On the other hand, quercetin may possess potent antioxidant activity, as demonstrated in animal studies [73]. Moreover, studies using in vitro models demonstrated that quercetin attenuated the expression of the inflammatory cytokines TNF-α, IFN-γ, IL-6, and IL-1β transcripts in cultured human macrophages [74]. Human studies using a prolonged endurance exercise model (i.e., treadmill running or cycling) reported that chronic (3 weeks) pure quercetin supplementation did not protect against exercise-induced oxidative stress and inflammation [75]. However, when quercetin was consumed for 2 weeks alongside the co-ingestion of other components (e.g., epigallocatechin 3-gallate), supplementation was found to counteract inflammation [76]. In contrast, even if quercetin was combined with other components, acute (15 min before exercise) supplementation did not attenuate post-exercise inflammation [77]. Thus, the effect of quercetin appears to vary depending on the duration of intake and/or if another nutritional supplement is co-ingested. More recently, a study testing triathletes demonstrated that a quercetin supplement designed especially to increase quercetin bioavailability may reduce oxidative stress (i.e., d-ROMs) and muscle pain immediately after training, with an improvement in the total time in all the three single events (swim, bike and run) simulating a triathlon race [78]. Thus, any approach that increases the bioavailability of quercetin is important for increasing its effectiveness. Previous paralleled and crossover design studies that examined the effect of quercetin on EIMD and DOMS markers are summarized below (Table 4).

### 5.1. Paralleled Design Studies 

Askari et al. (2012) showed that an 8-week supplementation of 500 mg/day quercetin combined with 200 mg/day vitamin C reduced plasma CK activity in male students [79]. In addition, Martin-Rincon et al. (2020) reported that a single dose of 140 mg mango leaf extract (Zynamite^®^) combined with 140 mg quercetin ingested one hour before 10-km running competition plus 100 drop jumps, followed by three additional doses (every 8 h thereafter for 24 h) attenuated muscle pain and the loss of jumping performance 24 h later compared with the placebo ingestion group in physically active male and female students [80]. In this study, the increases in the muscle damage marker in blood (Mb) following exercise were attenuated by quercetin supplementation in males only. Although CRP increased 24 h after exercise, quercetin supplementation had no effect on CRP at any point. On the other hand, O’Fallon et al. (2012) reported that 1000 mg/day (for 7 days before and 5 days after exercise) quercetin supplementation had no effect on markers of muscle damage (CK, muscle strength, soreness, resting arm angle, and upper arm swelling) or inflammation (IL-6 and CRP) after 24 eccentric exercises of the elbow flexors [81].

### 5.2. Crossover Design Studies

Bazzucchi et al. (2019) demonstrated the effect of 14-day quercetin supplementation (1000 mg/day) on neuromuscular impairment before, during, and after eccentric exercise of the elbow flexors in young active males [82]. Before exercise, quercetin supplementation increased isometric strength during MVIC compared to the baseline. During the eccentric exercise, the torque and muscle fiber conduction velocity decay were smaller in quercetin than in placebo ingestion. Immediately after exercise, isometric strength, the force–velocity relationship, and muscle fiber conduction velocity were lower in the placebo condition than in the quercetin ingestion condition. The authors concluded that quercetin supplementation seems to attenuate the severity of muscle weakness by sarcolemmal action potential propagation impairment [82]. A subsequent study by the same group using a similar design (the same quercetin dose, duration, and exercise protocol) demonstrated that 14-day quercetin supplementation (1000 mg/day) attenuated the increase in biomarkers of muscle damage (CK and LDH) associated with eccentric exercise [83]. Regarding these results, the authors speculated that 14 days of quercetin supplementation might have reduced lipid peroxidation and improved the redox status, ultimately increasing membrane resistance to mechanical stress [83].

**Table 4:** Effect of quercetin on EIMD and DOMS markers.

Reference (Year)	Population	Supplementation		Exercise	Outcome				
		Dose	Duration		Blood Damage Maker	Functional Performance Marker	DOMS, Pain	Inflammatory Marker	Oxidative Stress Marker
*Paralleled design studies*									
Askari et al. (2012) [79]	Male students	500 mg/day of quercetin with or without 200 mg/day vitamin C or placebo	8 weeks		CK: ◯ (quercetin + itamin C)	Time to exhaustion: ✕			
					AST: ✕				
O’Fallon et al. (2012) [81]	Healthy subjects, males and females	1000 mg/day of quercetin or placebo	12 d (7 d pre- and 5 d post-Ex)	Eccentric Ex (elbow flexors)	CK: ✕	Muscle strength, ROM, Swelling: ✕	VAS: ✕	IL-6, CRP: ✕	
Martin-Rincon et al. (2020) [80]	Physically active students, males and females	140 mg of quercetin with 140 mg of Zynamite^®^ or placebo	2 d (Pre-Ex, and every 8 h for 24 h)	Ran a 10-km race followed by 100 drop jumps	Mb: ◯ (males)	CMJ, mechanical impulse: ◯	VAS: ◯	CRP: ✕	
					CK: ✕				
*Crossover design studies*									
Bazzucchi et al. (2019) [82]	Moderately active males	500 mg of quercetin or placebo, twice/day	14 d (Pre-Ex)	Eccentric Ex (elbow flexors)	CK, LDH: ◯	FV, MVIC, MFCV, ROM: ◯	VAS: ✕		
						Circumference: ✕			
Bazzucchi et al. (2020) [83]	Low-to-moderate physically activate males	500 mg of quercetin or placebo, twice/day	14 d (Pre-Ex)	Eccentric Ex (elbow flexors)	CK, LDH: ◯	FV, MVIC, MFCV, ROM: ◯	VAS: ✕		
						Circumference: ✕			

◯, effective; ✕, ineffective; DOMS, delayed-onset muscle soreness; IL-6, interleukin-6; CRP, C-reactive protein; CK, creatine kinase; Mb, myoglobin; AST, aspartate aminotransferase; LDH, lactate dehydrogenase; MVIC, maximal voluntary isometric contraction; CMJ, counter movement jump; ROM, range of motion; FV, force–velocity relationship; MFCV, muscle fiber conduction velocity; VAS, visual analogue scale; PLA, placebo; Ex, exercise.

### 5.3. Summary

Quercetin ingestion does not provide anti-inflammatory effects after neither endurance nor local (e.g., elbow flexors and drop jumps) exercise in a parallel study design [80,81]. No crossover study has been conducted regarding the anti-inflammatory effects of quercetin. Several studies in which muscle damage was not assessed reported that quercetin supplementation enhances endurance exercise performance or anaerobic capacity as a result of improvement of mitochondrial biogenesis and antagonizing adenosine receptors [84,85]. In addition, quercetin may attenuate muscle strength loss following eccentric exercise or running [80,82,83] regardless of the research design. This ergogenic effect of quercetin may reflect an improvement of action potential propagation impairment due to the fact that the Ca^2+^ released from the sarcoplasmic reticulum or a blocking effect on the adenosine receptors, which may influence motor unit recruitment capacity. Most previous studies reported that quercetin attenuates muscle damage as assessed by blood markers (CK, LDH, and Mb), regardless of the study design, and independent of the physical activity [79,80,82,83]. The effect of quercetin on DOMS has been shown to be effective in a parallel study only [80]; thus, future crossover studies are warranted. The timing of quercetin intake may need to be considered because of the short half-life of quercetin (3.5 h) [86]. In addition, a clear anti-inflammatory effect has been reported in studies wherein quercetin was co-ingested with isoquercetin, n-3 polyunsaturated fatty acids (eicosapentaenoic acid and docosahexaenoic acid), and epigallocatechin 3-gallate [76]. As described in the aforementioned studies, a decreasing effect of quercetin on blood markers of muscle damage (CK and Mb) was observed when quercetin was co-ingested with vitamin C [79] or mango leaf extract [80]. Therefore, to maximize the effect of quercetin, the ingestion of additional nutritional supplements or the timing of quercetin intake may be important.

## 6. Isothiocyanate

All of the supplements reviewed herein, namely curcumin, tart cherry juice, beetroot juice, and quercetin, were classified as phenolic compounds. On the other hand, isothiocyanate, which is classified as an organosulfur compound, is an emerging phytochemical [87]. Isothiocyanate is found in vegetables, including those of the *Brassica* (Cruciferous) genus. For example, benzyl isothiocyanate and phenethyl isothiocyanate are found in cabbage and watercress, whereas sulforaphane is found in broccoli. Allyl isothiocyanate and 6-methylsulfinylhexyl isothiocyanate (6-MSITC) are present in wasabi (*Wasabia japonica*) which is a typical Japanese pungent spice. These compounds have cardioprotective and anticarcinogenic effects [88]. The common actions of isothiocyanate in organosulfur compounds have anti-inflammatory and antioxidant effects [87,89,90]. Therefore, isothiocyanate supplementation is expected to accelerate the recovery of EIMD and DOMS. However, very limited research regarding the effectiveness of isothiocyanate in improving EIMD is available in animal studies. 6-MSITC, a type of isothiocyanate, is known to be a potent Nrf2 activator and suppresses all three MAPK pathways, exhibiting anti-inflammatory and antioxidant properties [91,92]. In addition to these characteristics, a previous work reported that 6-MSITC might suppress calpain-1 activation, which is a Ca^2+^-dependent protease [93], in the muscle tissue. Moreover, the same study demonstrated that 6-MSITC administration attenuates CK activity after forced swimming in mice [94]. The inhibition of calpain activity accelerates the force production restoration process after eccentric contractions in rats [95]. Based on these results obtained in animal studies, 6-MSITC intake is expected to accelerate the recovery process of reduced maximum muscle contraction after eccentric exercise in humans. A pilot study using a randomized, double-blind, crossover design examined the effect of 5-day 6-MSITC supplementation (9 mg/day) on EIMD and DOMS after eccentric exercise of the elbow flexors in young active males. In contrast to the hypothesis, calpain-1, muscle damage (MVIC torque, ROM, DOMS, CK, and swelling), and inflammatory markers (IL-8 and TNF-α) were not affected by 6-MSITC relative to those in the placebo-treated condition [96]. Given that this is the only human study assessing the effect of 6-MSITC, more human studies are needed to delineate the effectiveness of isothiocyanate in humans in the future.

## 7. Conclusions

### 7.1. Remarks

In the current review, dietary supplements with anti-inflammatory and antioxidant effects are discussed. Some positive effects mediated by curcumin, tart cherry juice, beetroot juice, and quercetin have been reported in EIMD and DOMS, although some of these results are not consistent among previous studies. These supplements may not only attenuate the aggravation of secondary muscle damage, but also improve performance by modulating cardiorespiratory and neuromuscular efficiency possibly in an interactive manner. It should be highlighted that exercise modality, physical fitness level, and study design need to be considered when interpreting the results of supplementation effects. Furthermore, the dose and duration of supplementation are important factors to maximize the effect of supplementation on EIMD and DOMS.

### 7.2. Future Perspectives

When using dietary supplements in competition or daily training to attenuate EIMD or DOMS, it is advisable for all individuals, including athletes, coaches, and experts, to interpret research outcomes. The dietary supplements presented in this review are included in the IOC consensus statements for high-performance athletes; however, the evidence is still limited and not well established for EIMD and DOMS. Moreover, we were unable to determine an appropriate dose and duration for each supplement for attenuating EIMD and DOMS, as the bioavailability and half-life of supplements can vary depending on their purification methods and forms. More studies are required to draw a firm conclusion regarding appropriate dose and duration. Moreover, further research is needed to identify the effectiveness of dietary supplements for EIMD and DOMS, especially in elite athletes [97].

In addition, we need to understand the differences between natural vs. purified products. We may need to take a large amount of natural products to increase the bioavailability necessary to attenuate EIMD and DOMS. It is also important to note that natural products may contain non-target ingredients which might modulate the action of supplements. Therefore, it is necessary to pay attention not only to the amount but also to the form of products.

## Figures and Tables

**Table 1:** Effect of curcumin on EIMD and DOMS markers.

Reference (Year)	Population	Supplementation		Exercise	Outcome				
		Dose	Duration		Blood Damage Maker	Functional Performance Marker	DOMS, Pain	Inflammatory Marker	Oxidative Stress Marker
*Paralleled Design Studies*									
Drobnic et al. (2014) [33]	Healthy, moderately active males	200 mg of curcumin or placebo, twice/day	4 d (2 d pre- and 2 d post-Ex)	Downhill run	CK: ✕		VAS: ◯	IL-8: ◯	FRAT, CAT, GPx: ✕
								CRP, MCP-1: ✕	
Tanabe et al. (2019) [34]	Healthy young males	PRE, POST: 90 mg of curcumin, twice/day	PRE: 7 d pre-Ex	Eccentric Ex (elbow flexors)	CK: ✕	ROM: ◯ (POST)	VAS: ◯ (POST)		
			POST: 4 d post-Ex			ROM: ✕ (PRE)			
		PLA: 90 mg of placebo, twice/day					VAS: ✕ (PRE)		
			CON: 4 d post-Ex			MVIC: ✕			
Faria et al. (2020) [35]	Healthy normal-weight males	500 mg of curcumin or placebo, three times/day	29 d	Half-marathon	Mb: ◯			IL-10: ◯	
					CK, LDH, AST: ✕			IL-6: ✕	
*Crossover Design Studies*									
Tanabe et al. (2015) [31]	Untrained young males	150 mg of curcumin or placebo	1 h pre- and 12 h post-Ex	Eccentric Ex (elbow flexors)	CK: ◯	MVIC: ◯	VAS: ✕	IL-6, TNF-α: ✕	
						ROM, swelling: ✕			
Nicol et al. (2015) [36]	Physically active males	2.5 g/day of curcumin or placebo, twice/day	5 d (2.5 d pre- and 2.5 d post-Ex)	Eccentric Ex (single-leg press)	CK: ◯	Jump performance: ◯	VAS: ◯	IL-6: ◯ TNF-α: ✕	
						Swelling: ✕			
Delecroix et al. (2017) [30]	Male elite rugby players	2 g of curcumin + 20 mg of piperine, or placebo, three times/day	4 d (2 d pre- and 2 d post-Ex)	Single leg jumps on an 8% downhill slope	CK: ✕	Sprint: ◯	VAS: ✕		
Tanabe et al. (2019) [37] Experiment 1	Healthy males	90 mg of curcumin or placebo, twice/day	7 d pre-Ex	Eccentric Ex (elbow flexors)	CK: ✕	MVIC, ROM: ✕	VAS: ✕	IL-8: ◯	d-ROMs, BAP: ✕
						Swelling: ✕		TNF-α: ✕	
Tanabe et al. (2019) [37] Experiment 2	Healthy males	90 mg of curcumin or placebo, twice/day	7 d post-Ex	Eccentric Ex (elbow flexors)	CK: ◯	MVIC, ROM: ◯	VAS: ◯	IL-8: ✕	d-ROMs, BAP: ✕
						Swelling: ✕		TNF-α: ✕	

◯, effective; ✕, ineffective; DOMS, delayed-onset muscle soreness; IL-6, interleukin-6; IL-8, interleukin-8; IL-10, interleukin-10; TNF-α, tumor necrosis factor-α; CRP, C-reactive protein; MCP-1, monocyte chemoattractant protein 1; CK, creatine kinase; Mb, myoglobin; LDH, lactate dehydrogenase; AST, aspartate aminotransferase; MVIC, maximal voluntary isometric contraction; ROM, range of motion; VAS, visual analogue scale; FRAP, ferric reducing ability plasma; CAT, catalase; GPx, glutathione peroxidase; d-ROMs, diacron-reactive oxygen metabolites; BAP, biological antioxidant power; PLA, placebo; PRE, pre-exercise supplementation; POST, post-exercise supplementation; Ex, exercise.

**Table 2:** Effect of tart cherry juice on EIMD and DOMS markers.

Reference (Year)	Population	Supplementation		Exercise	Outcome				
		Dose	Duration		Blood Damage Maker	Functional Performance Marker	DOMS, Pain	Inflammatory Marker	Oxidative Stress Marker
*Paralleled design studies*									
Howatson et al. (2010) [39]	Recreational marathon runners, males and females	236 mL TCJ or placebo, twice/day	8 d (5 d pre-Ex, Ex-d, and 2 d post-Ex)	Marathon	CK, LDH: ✕	MVIC: ◯	VAS: ✕	IL-6, CRP, Uric Acid: ◯	TAS, TBARS: ◯
									PC: ✕
Bell et al. (2016) [48]	Semi-professional male soccer players	30 mL TCJ or placebo, twice/day	8 d (4 d pre-Ex, Ex-d, and 3 d post-Ex)	LIST	CK: ✕	MVIC, CMJ, agility: ◯	VAS: ◯	IL-6: ◯	LOOH: ✕
						Sprint: ✕		IL-8, IL-1-β CRP, TNF-α: ✕	
Quinlan et al. (2019) [49]	Team-sport players, males and females	30 mL TCJ or placebo, twice/day	8 d (5 d pre- Ex, Ex-d, and 2 d post-Ex).	LIST	CK: ✕	MVIC, CMJ, sprint: ◯	VAS: ✕	CRP: ✕	
Lamb et al. (2019) [50]	Non-resistance trained males	TCJ: 30 mL TCJ, twice/day	9 d (4 d pre-Ex, Ex-d, and 4 d post-Ex)	Eccentric Ex (elbow flexors)	CK: ✕	MVIC, ROM: ✕	VAS: ✕		
		POM: 250 mL of pomegranate juice, twice/day							
		PLA: placebo drink, twice/day							
*Crossover design studies*									
Connolly et al. (2006) [51]	Male college students	355 mL TCJ or placebo, twice/day	8 d (4 d pre-Ex, Ex-d, and 3 d post-Ex)	Eccentric Ex (elbow flexors)		MVIC: ◯	VAS: ◯		
						ROM: ✕			
Bowtell et al. (2011) [52]	Well-trained males	30 mL TCJ or placebo, twice/day	10 d (7 d pre-Ex, and 2 d post-Ex)	Single-leg knee extensions at 80% 1RM	CK: ✕	MVIC: ◯	PPT: ✕	CRP: ✕	Nitrotyrosine, TAS: ✕
									PC: ◯
Morehen et al. (2020) [53]	Professional male rugby players	30 mL TCJ or placebo, twice/day	8 d (5 d pre-Ex, Ex-d and 2 d post-Ex)	Rugby match		CMJ, drop jump: ✕	VAS: ✕	IL-6, IL-8, IL-10: ✕	
Abbott et al. (2020) [54]	Professional male soccer players	30 mL TCJ or placebo, twice/day	3 d (pre- and post-Ex and 12 and 36 h post- Ex)	90-min soccer match		CMJ, reactive strength: ✕	VAS: ✕		

◯, effective; ✕, ineffective; DOMS, delayed-onset muscle soreness; IL-1-β, interleukin-1-beta; IL-6, interleukin-6; IL-8, interleukin-8; IL-10, interleukin-10; TNF-α, tumor necrosis factor-α; CRP, C-reactive protein; CK, creatine kinase; LDH, lactate dehydrogenase; MVIC, maximal voluntary isometric contraction; ROM, range of motion; CMJ, counter movement jump; VAS, visual analogue scale; PPT, pressure pain threshold; TAS, total antioxidant status; TBARS, thiobarbituric acid reactive substances; PC, protein carbonyls; CAT, catalase; GPx, glutathione peroxidase; LOOH, lipid hydroperoxides; PLA, placebo; TCJ, tart cherry juice; POM, pomegranate juice; Ex, exercise; 1RM; 1-repetition maximum; LIST, Loughborough intermittent shuttle test.
